# Characterization of Cold and Heat Tolerance of *Bactrocera tau* (Walker)

**DOI:** 10.3390/insects13040329

**Published:** 2022-03-28

**Authors:** Huan Liu, Xiaoyan Wang, Zihan Chen, Yongyue Lu

**Affiliations:** 1 College of Horticulture and Plant Protection, Henan University of Science and Technology, Luoyang 471023, China; c16691386863@163.com; 2 Key Laboratory of South Subtropical Fruit Biology and Genetic Resource Utilization (MOA), Institute of Fruit Tree Research, Guangdong Academy of Agricultural Sciences, Guangzhou 510642, China; 3 Department of Entomology, South China Agricultural University, Guangzhou 510642, China; wxiaoyan202201@126.com

**Keywords:** *Bactrocera tau*, supercooling point, cold hardiness, heat tolerance, ULCIZ, LLTIZ

## Abstract

**Simple Summary:**

Insects are often stressed by adverse factors in their natural environment. Temperature is a crucial driver of insect activity, adaptability, and distribution, and therefore, it greatly impacts the invasive success of alien pests. *Bactrocera tau* (Walker) is an invasive polyphagous herbivore of vegetables and fruits, now a pest of global importance. This study provides useful information about *B. tau*’s cold- and heat tolerance to extremely low and high temperatures. Its different life stages (i.e., egg, larvae, pupae, and adult) had high survival rates under adverse temperatures spanning −5 to 0 °C and 39 to 42 °C. These findings suggest that *B. tau* possesses a wide temperature threshold range for survival, which likely contributes to its better establishment and expansion in new regions. Meanwhile, fitted curves were used to quantify *B. tau*’s tolerance potential as a function of both stress intensity (heat or cold) and exposure duration. The information generated in this study will contribute to our understanding of thermal tolerance in *B. tau* and could also provide insights for devising phytosanitary control approaches.

**Abstract:**

*Bactrocera tau* (Walker) (Diptera: Tephritidae) is a serious, economically important invasive pest that has spread and been established in many regions worldwide. Temperature is a crucial abiotic factor governing insect activity, fitness, and geographical distribution. Yet, surprisingly, the tolerance of *B. tau* to extreme cold and heat stress remains unclear. Here, we measured the supercooling point (SCP) of different life stages of *B. tau*. Further, several life stages of *B. tau* (egg, 1st, 2nd, and 3rd instar larvae, 1-day-old pupae, and 3-day-old adult) were subjected to six low temperatures (−9, −7, −5, −3, −1, and 0 °C) and six high temperatures (39, 40, 41, 42, 43, and 44 °C) for various durations (0.5, 1.0, 2.0, and 4.0 h), and three-way survival–time–temperature relationships were investigated. We found that the SCPs differed significantly among different life stages of *B. tau*, being the lowest for SCP of eggs, at −25.82 ± 0.51 °C. There was no significant effect of sex on the mean SCPs of *B. tau* adults, except for 45- to 50-day-old flies. In addition, an interaction effect was uncovered between tested temperatures and exposure duration upon *B. tau* mortality at different life stages. Eggs exhibited the strongest cold tolerance, yet the weakest heat tolerance. The 3rd instar larvae were the most heat- and cold tolerant among larval stages, followed by the 2nd and 1st instar larvae. The upper limit of the chill injury zone (ULCIZ) for 3-day-old adult and 1-day-old pupae was −2.51 °C and −2.50 °C, respectively, while their corresponding lower limit of thermal injury zone (LLTIZ) was 39.39 °C and 38.29 °C. This paper presents valuable data to provide an integrated knowledge for understanding the cold and heat tolerance potential of *B. tau* and ensure the proper implementation of post-harvest phytosanitary protocols for this pest’s disinfestation.

## 1. Introduction

The pumpkin fruit fly, *Bactrocera tau* (Walker) (Diptera: Tephritidae), is an economically dangerous invasive pest that attacks many agricultural crops and is now distributed throughout many regions worldwide [1,2,3]. It is holometabolous that goes from egg, larva, pupa before reaching adulthood and reproductive maturity. A short life cycle (approximately 15 days) means a high number of generations overlapping per year under optimal conditions [1,4]. In most areas of China, *B. tau* can complete 3–8 generations annually, and it has no obligatory diapausing stage while it survives the winter as pupae in soil or damaged fruits [4,5,6]. Native to Fujian province in China in 1849, this fly quickly spread beyond its native range throughout much of southern China around 1912; it is generally present in South Asia, Southeast Asia, East Asia, sub-Equatorial Africa, Australia, Solomon Islands, as well as the South Pacific region from 2000 [3,7,8]. It is well known that *B. tau* can severely damage more than 80 different cultivars of fruit and vegetables, e.g., pumpkin, luffa, cucumber, melon, eggplant, tomato, mango, orange, papaya, etc., [1]. The process and pattern of herbivory by *B. tau* is similar to that of other fruit fly species. Female flies use a sclerotized ovipositor to pierce through the fruit skin, laying their eggs inside it; newly hatched larvae feed inside the fruit pulp until they mature, after which the infested crop quickly becomes rotten and drops to the ground prematurely [9]. Because it is able to invade rapidly in areas with favorable environmental conditions, while simultaneously disrupting the agricultural production and international trade, *B. tau* is designated a serious economic pest and is on the quarantine lists of many countries, e.g., Japan, Korea Rep, Indonesia, Pakistan, Jordan, the United States, etc., [2,7].

Insects are typical poikilothermous animals, in that their body temperature is profoundly affected by the ambient climate [10,11]. Temperature is a key parameter that strongly determines the dynamics of insect survival, abundance, and geographical distribution [3,12,13,14]. All insects have a specific range of temperatures within which they can grow and reproduce [15]. Too-high temperatures may positively or negatively impact their mating, oviposition, and thermotactic behavior [16], while low temperature stress may inhibit the activity of bio-macromolecules in vivo, reduce rates of metabolism, affect both development and reproduction, and even disturb the sex ratio balance of insects [17]. In general, cold and heat tolerance are important eco-physiological traits related directly to the fitness, survival, and distribution of invasive insects [11,18,19]. Notably, cold tolerance is a vital strategy to prevent cold injuries in insects during harsh winter conditions [13,14,19]. The supercooling point (SCP), defined as the temperature at which point an insect’s body fluids spontaneously freeze, is a key indicator for evaluating the cold tolerance of many insect species [11,13,14,20,21,22]. The SCP is predetermined by the insect’s inherent biological characteristics; these are closely linked to their body composition and physiological status, e.g., feeding stage, diapause, life stage, and overwintering by freezing avoidance [23]. 

In particular, extreme temperatures can be a critical factor governing insects’ population dynamics [24,25]. In this context, extreme high and low temperatures determine the geographic range expansion of invasive insects at different latitudes, altitudes, and across landscapes which define their appropriate temperature threshold range [26]. Accordingly, studies investigating the tolerance ability of insects to extremely high and low temperatures have become more prominent in defining the extent of their distribution in a given country or region, in the successful forecasting of their potential range, and in controlling and preventing pest outbreaks [14,27]. Hence, in this study, our objectives were to determine (1) the supercooling capacity of *B. tau*, (2) it’s tolerance of extreme cold, and (3) it’s tolerance of extreme heat, as well as (4) the upper limit of the chill injury zone (ULCIZ) and lower limit of the thermal injury zone (LLTIZ) for several key life stages of *B. tau*. This paper’s findings will contribute to our understanding of cold- and heat-tolerance strategies in *B. tau*. Furthermore, the information and data generated in our study may prove useful to devising phytosanitary measures required for the quarantine treatment of *B. tau*.

## 2. Materials and Methods

### 2.1. Insect Rearing

The pumpkin fruit fly was maintained under laboratory conditions at the South China Agricultural University, Guangzhou, China, and reared at 25 ± 1 °C, under 75 ± 1% relative humidity and a photoperiod of 14-h:10 h (light: dark). The *B. tau* populations were reared for at least 20 generations. Their eggs were collected using cucumber slices, and hatched larvae reared on pumpkin. Adult flies were reared in mesh cages (60 cm × 60 cm × 60 cm) and fed an artificial diet consisting of yeast extract: dry sugar in a 1:3 ratio (*w*/*w*) and water. 

### 2.2. Measuring the SCPs of B. tau at Different Life Stages

To measure the SCPs of *B. tau* at different life stages, a thermocouple (NiCr-Ni probe) connected to an automatic temperature recorder (Testo, Friedrichshafen, Germany) was used according to published procedures [11,14,18]. Individuals of different developmental stages were randomly selected for testing, namely the eggs, 1st to 3rd instar larvae, 1- to 8-day-old pupae, and 2- to 3-day-old adults, 8- to 10-day-old adults, 25- to 30-day-old adults, 45- to 50-day-old adults, 65- to 70-day-old adults, 85- to 90-day-old adults, and 105- to 110-day-old adults. Using plastic glue tape, these test individuals (*n* = 30 samples per stage) were attached to the thermocouple (NiCr-Ni probe) and placed inside a programmable refrigerated chamber (Binder GmbH Bergstr, Model MK 57, Tuttlingen, Germany). The temperature fell from 10 °C to −30 °C at a rate of 0.5 °C/min. The data were read using Comsoft 3 Software. The temperature at which a sudden increase occurred due to the liberated latent heat of freezing was recorded as the SCP of each individual insect. After every such treatment, the thermal probe was calibrated with an ice water mixture. Ten individual insects were processed in each treatment run, with the treatment repeated three times per stage.

### 2.3. Determining the Tolerance of B. tau to Extreme High and Low Temperatures

This experiment was performed by following established methods, albeit slightly modified [12,14,28,29,30,31]. The cold tolerance response of *B. tau* was tested using its exposure to −9, −7, −5, −3, −1, and 0 °C, whereas its heat tolerance was assessed against 39, 40, 41, 42, 43, and 44 °C temperatures; the exposure duration to each temperature treatment was 0.5, 1.0, 2.0, or 4.0 h. Temperature treatment at 25 °C was used as the control. The eggs, the 1st, 2nd, and 3rd instar larvae, 1-day-old pupae, and 3-day-old adults (sex ratio, male to female = 1:1), which had been kept at room temperature (25 °C), were randomly selected and allocated to each treatment. Each test sample (*n* = 30) was put into a round bottom glass tube (30 mm × 200 mm) and this was sealed with a breathable film. When the thermostatic bath reached the required temperature, all the glass tubes were quickly placed in it. After this treatment, the eggs were transferred onto the surface of an artificial diet in a separate environmental chamber (25 °C) and allowed to hatch, while the pupae were embedded in sand 2–3 cm under 10% humidity at room temperature (25 °C) and allowed to emerge; subsequently, their egg hatching rate and pupal emergence rate were recorded. For the treated larvae and adults, their respective survivorship was recorded after 20 min of recovery. When touched with a brush, larvae and adults who showed no movement were recorded as dead. There were 30 individuals in each replicate and control group. Each experiment consisted of three replicates.

### 2.4. Evaluating the Relationship of Temperature–Time–Mortality in B. tau

The ability of *B. tau* to resist temperature stress was evaluated by applying corrected mortality (Formula (1)). In order to systematically explore the interactive effect of treatment temperature (*T*) and exposure time (*t*) on the mortality rate (*S*) of *B. tau* at its different life stages, a bivariate logistic equation method was used to fit a three-dimensional data curve (Formulas (2) and (3)) [19,28,32].

(1) $$\mathrm{Corrected}\;\mathrm{mortality}\;\mathrm{rate}\;(S,\;\%)\;=\;\frac{\mathrm{Mortality}\mathrm{of}\mathrm{treatment}\mathrm{group}\;-\;\mathrm{Mortality}\;\mathrm{of}\;\mathrm{control}\;\mathrm{group}}{1\;-\;\mathrm{Mortality}\;\mathrm{of}\;\mathrm{control}\;\mathrm{group}}\;\times \;100$$

*S* = *e*^(*a*+*b*×*t*×(*T*−*c*))^/(1 + *e*^(*a*+*b*×*t*×(*T*−*c*))^) (2)

*S* = *e*^(*a*+*b*×*t*×(*C*−*T*))^/(1 + *e*^(*a*+*b*×*t*×(*C*−*T*))^) (3)

where *S* represents the corrected mortality rate, *T* and *t* represent temperature and time, respectively; *a*, *b* are constant parameters; *c* is an estimate of the ULCIZ, this being the temperature that begins to cause death under continuous cold stress; *C* is an estimate of the LLTIZ, the temperature above which mortality occurs under continuous heat stress. The unit of *C* and *c* are degrees Celsius (°C). These parameters were used to draw the three-dimensional surface plots.

### 2.5. Data Analysis

All data from experimental replicates were expressed as the mean ± standard error, and all statistical analyses were carried out using SAS 9.20 software (SAS Institute Inc., Cary, NC, USA). Data for each response variable were normally distributed and had similar variance after checking with the Shapiro–Wilk test and Levene’s test, respectively. The SCP of different life stages of *B. tau*, and their corrected mortality under different temperature treatments, were compared using one-way ANOVA (followed by Duncan’s multiple range test, DMRT) or two-way ANOVA (followed by Tukey’s multiple comparison post hoc test). For these ANOVAs, data were first converted to homogenize the variances (*p* < 0.05). LLT_50_ was calculated to evaluate the damage to insects caused by low and high temperatures. SAS software was also used for the curve fitting exercises. All results were plotted in Origin 9.0 software.

## 3. Results

### 3.1. The SCPs of B. tau at Different Life Stages

The SCPs were different among various life stages of *B. tau* (Table 1). The lowest SCP was recorded for eggs (−25.82 ± 0.51 °C) followed by 6-day-old pupae (−22.95 ± 0.34 °C). The SCPs of 1st instar larvae were respectively slightly higher than that of 2nd and 3rd instar larvae. Furthermore, the SCPs tended to decrease with pupal age; those of 1-day-old pupae (−9.77 ± 0.49 °C) were evidently higher than those of 6-day-old pupae (−22.95 ± 0.34 °C). Moreover, the SCPs tended to decrease at first, then increase, and finally stabilize as adult age increased. The 8- to 10-day-old male adults had the lowest SCPs at −15.69 ± 0.90 °C. Furthermore, the 45- to 50-day-old female adults had the highest SCPs, at −7.69 ± 0.72 °C. In addition, SCPs were similar between the adulthood sexes at the same developmental stage, except for the 45- to 50-day-old adults.

### 3.2. Cold Tolerance of B. tau at Different Developmental Stages

**Figure 1:**
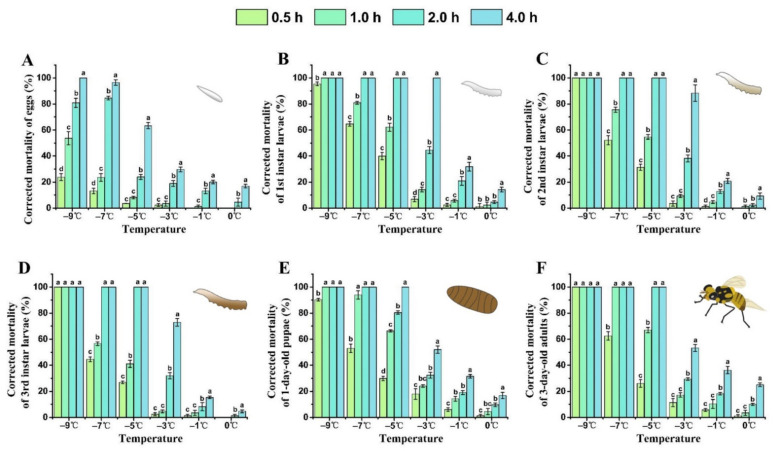
Influence of extreme low temperature and exposure time on the mortality rate of different developmental stages of *Bactrocera tau*. (**A**) Eggs, (**B**) 1st instar larvae, (**C**) 2nd instar larvae, (**D**) 3rd instar larvae, (**E**) 1-day-old pupae, and (**F**) 3-day-old adults. Bars represent mean ± SE values. Different letters denote significant differences among exposure times at the same treatment temperature (ANOVA, *p* < 0.05).

With a lower treatment temperature and longer exposure time, the corrected mortality of each developmental stage of *B. tau* gradually increased (Figure 1). The semi-lethal temperature (LLT50) of all stages of *B. tau* increased with the extension of exposure time (Table 2). For all stages, mortality was lower than 10% after their acute exposure to 0 °C for 0.5–2.0 h, whereas, after 4 h exposure to this temperature, the corrected mortality of eggs, 1st instar larvae, 1-day-old pupae, and 3-day-old adults respectively reached 16.84 ± 1.40%, 14.19 ± 1.80%, 16.82 ± 2.47%, and 25.04 ± 1.36%. The mortality rate of 3rd instar larvae after exposure to cold at −7 °C for 1 h was 56.56 ± 1.39%; this was significantly lower than that for 1st larvae (80.92 ± 1.19%) or 2nd larvae (75.62 ± 1.90%) (*F* = 50.92, *df* = 2, *P* = 0.0014). The corrected mortality rate of 1-day-old pupae and 3-day-old adults was 53.03 ± 3.34% and 62.54 ± 3.24%, respectively, after their exposure to −7 °C for 0.5 h, and that of 1-day-old pupae was 90.26 ± 0.98% after exposure to cold at −9 °C for 0.5 h. Nonetheless, each life stage reached 100% mortality after acute exposure to cold at –9 °C for 4 h. At 4 h exposure, −2.40 °C caused 50% mortality in eggs and −0.86 °C caused 50% mortality in 1st instar larvae. The results indicated that 1st instar larvae were the most sensitive stage to cold, and eggs were the most cold-tolerant stage of insect development in *B. tau*.

### 3.3. Interaction between Temperature Exposure Duration and Low Temperature for Mortality of B. tau at Different Developmental Stages

**Figure 2:**
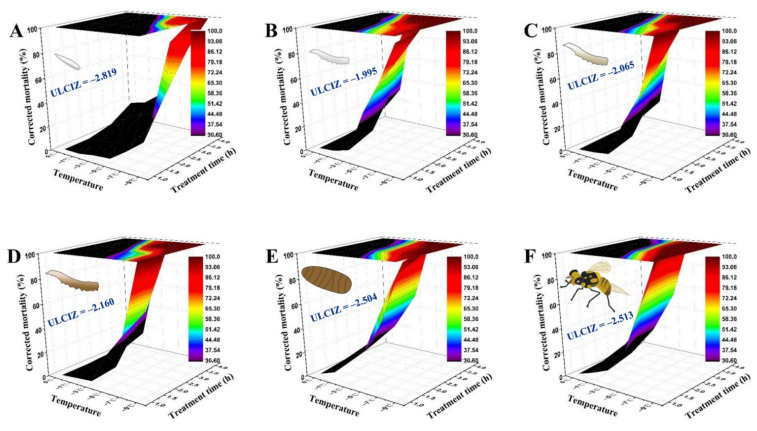
Three-dimensional surface models describing the relationship between exposure time, low temperature, and corrected mortality of different developmental stages of *Bactrocera tau*. The upper limit of the chill injury zone (ULCIZ) corresponds to the temperature that begins to cause death under continuous cold stress. (**A**) Eggs (ULCIZ = −2.819 °C), (**B**) 1st instar larvae (ULCIZ = −1.995 °C), (**C**) 2nd instar larvae (ULCIZ = −2.065 °C), (**D**) 3rd instar larvae (ULCIZ = −2.160 °C), (**E**) 1-day-old pupae (ULCIZ = −2.504 °C), and (**F**) 3-day-old adults (ULCIZ = −2.513 °C).

As Figure 2 shows, the temperature–time–mortality curves of the eggs, the 1st, 2nd, and 3rd instar larvae, 1-day-old pupae, and 3-day-old adults were successfully fitted by the extended logistic equation, *S* = *e*^(*a*+*b*×*t*×(*T*−*c*))^/(1 + *e*^(*a*+*b*×*t*×(*T*−*c*))^). Its estimated values were as follows: *a* = −2.612, *b* = −0.331, *c* = −2.819 for eggs (*R^2^* = 0.955, *F* = 147.22, *p* < 0.0001); *a* = −2.669, *b* = −1.258, *c* = −1.995 for 1st instar larvae (*R^2^* = 0.978, *F* = 208.86, *p* < 0.0001); *a* = −3.001, *b* = −1.288, *c* = −2.065 for 2nd instar larvae (*R^2^* = 0.983, *F* = 393.70, *p* < 0.0001); *a* = −2.792, *b* = −1.083, *c* = −2.160 for 3rd instar larvae (*R^2^* = 0.970, *F* = 225.70, *p* < 0.0001); *a* = −1.684, *b* = −0.901, *c* = −2.504 for 1-day-old pupae (*R^2^* = 0.977, *F* = 298.04, *p* < 0.0001); and *a* = −2.454, *b* = −1.358, *c* = −2.513 for 3-day-old adults (*R^2^* = 0.974, *F* = 265.08, *p* < 0.0001). Their corresponding graphs indicated that the survival of *B. tau* exposed to low temperature depends heavily on both the duration and temperature of exposure to cold. The ULCIZ (*c* value) of 3-day-old adults and 1-day-old pupae was −2.513 °C and −2.504 °C, respectively, both lower than that of 1st instar larvae (−1.995 °C).

### 3.4. Heat Tolerance of Different Developmental Stages of B. tau

**Figure 3:**
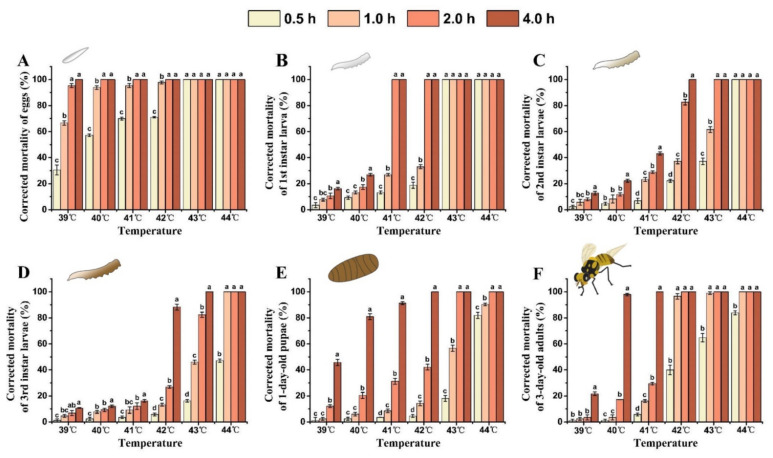
Effect of extreme high temperature and exposure time on the mortality rate of different developmental stages of *Bactrocera tau*. (**A**) Eggs, (**B**) 1st instar larvae, (**C**) 2nd instar larvae, (**D**) 3rd instar larvae, (**E**) 1-day-old pupae, and (**F**) 3-day-old adults. Bars represent mean ± SE values. Different letters denote significant differences among exposure times at the same treatment temperature (ANOVA, *p* < 0.05).

As both the treatment temperature and exposure time increased, so did the corrected mortality rate of each developmental stage of *B. tau* (Figure 3). The semi-lethal temperature (LLT50) of all stages of *B. tau* decreased with the extension of exposure time (Table 3). Eggs exhibited the lowest heat tolerance, having a corrected mortality rate of 95.30 ± 1.54% after exposure to 39 °C for 2 h. Mortality differed significantly between eggs and other life stages when exposed to 39 °C for 4 h, being 100%, 16.33 ± 1.04%, 12.82 ± 1.39%, 10.70 ± 0.34%, 45.72 ± 2.48%, and 21.63 ± 1.44% for the eggs, the 1st, and 2nd, and 3rd instar larvae, 1-day-old pupae, and 3-day-old adults, respectively (*F* = 611.12, *df* = 5, *p* < 0.0001). Eggs and 1st instar larvae were unable to survive at temperatures above 43 °C. Further, the 2nd and 3rd instar larvae, 1-day-old pupae, and 3-day-old adults all died after exposure to 43 °C for 4 h. The 3rd instar larvae, 1-day-old pupae, and 3-day-old adults showed stronger heat tolerance, and their corrected mortality rates after acute exposure to 44 °C for 0.5 h were 47.08 ± 1.32%, 81.87 ± 2.39%, and 83.95 ± 1.53%, respectively; however, all of them died after prolonged exposure (2 h) to heat. LLT_50_ of eggs and 3rd instar larvae were achieved at 27.35 °C and 40.74 °C, respectively, when exposed for 4 h. These results suggested the egg stage was the most susceptible to acute heat increases, but that 3rd instar larvae were the most heat-tolerant stage of development.

### 3.5. Interaction between Temperature Exposure Duration and High Temperature for Mortality of B. tau at Different Developmental Stages

**Figure 4:**
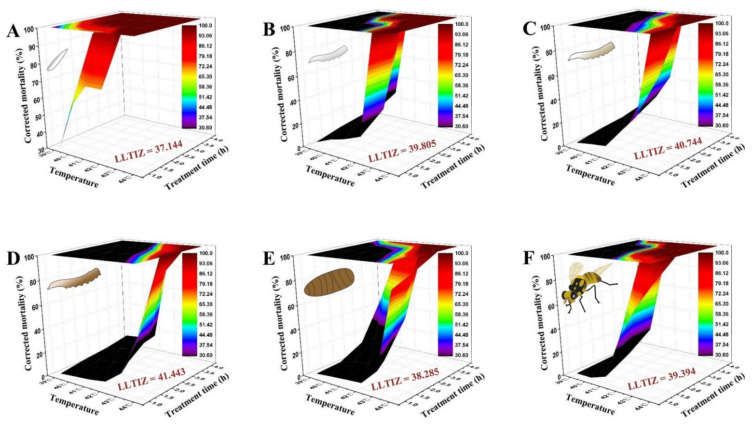
Three-dimensional surface models describing the relationship between exposure time, high temperature, and corrected mortality of different developmental stages of *Bactrocera tau*. The lower limit of the thermal injury zone (LLTIZ) corresponds to the temperature above which mortality occurs under continuous heat stress. (**A**) Eggs (LLTIZ = 37.144 °C), (**B**) 1st instar larvae (LLTIZ = 39.805 °C), (**C**) 2nd instar larvae (LLTIZ = 40.744 °C), (**D**) 3rd instar larvae (LLTIZ = 41.443 °C), (**E**) 1-day-old pupae (LLTIZ = 38.285 °C), and (**F**) 3-day-old adults (LLTIZ = 39.394 °C).

As depicted in Figure 4, the time–temperature–mortality curves of the eggs, the 1st, 2nd, and 3rd instar larvae, 1-day-old pupae, and 3-day-old adults were successfully fitted by the extended logistic equation. *S* = *e*^(*a*+*b*×*t*×(*C*−*T*))^/(1 + *e*^(*a*+*b*×*t*×(*C*−*T*))^), taking these values: *a* = −2.156, *b* = 1.553, *C* = 37.144 for eggs (*R^2^* = 0.998, *F* = 4463.80, *p* < 0.0001); *a* = −2.796, *b* = 2.043, *C* = 39.805 for 1st instar larvae (*R^2^* = 0.955, *F* = 147.57, *p* < 0.0001); *a* = −1.909, *b* = 1.517, *C* = 40.744 for 2nd instar larvae (*R^2^* = 0.966, *F* = 197.60, *p* < 0.0001); *a* = −3.203, *b* = 2.162, *C* = 41.443 for 3rd instar larvae (*R^2^* = 0.977, *F* = 297.91, *p* < 0.0001); *a* = −2.603, *b* = 0.517, *C* = 38.285 for 1-day-old pupae (*R^2^* = 0.910, *F* = 70.46, *p* < 0.0001); and *a* = −4.432, *b* = 2.537, *C* = 39.394 for 3-day-old adults (*R^2^* = 0.948, *F* = 128.47, *p* < 0.0001). The derived LLTIZ (*C* values) were evidently different among the developmental stages. The parameter analyses demonstrated that the heat tolerance of 3rd instar larvae was the strongest (LLTIZ = 41.443 °C), followed by that of 3-day-old adults (LLTIZ = 39.394 °C), 1-day-old pupae (LLTIZ = 38.285 °C), and eggs (LLTIZ = 37.144 °C).

## 4. Discussion

*Bactrocera tau* is a globally invasive polyphagous fruit fly pest, one capable of causing extensive damage to many cultivars of vegetable and fruit products [3]. It is regarded as an economically important pest and has been listed internationally as a quarantine organism [2]. In their natural environment, insects can incur stress from adverse abiotic factors, of which temperature is known to directly constrain the survival, geographic distribution, and spread of invasive pests in general [3,12,18,29]. Notably, assessing the impact of extreme temperature exposure on a given insect species is crucial to understanding its adaptability, population dynamics, and distributions, as well as for facilitating the management of alien (non-native) species [15]. However, the tolerance responses to acute cold and heat in the *B. tau* have not been reported in the literature. In this study, we found that the egg stage was the most resistant life stage to low temperatures, but sensitive to high temperatures. The 3rd instar larvae were the most heat and cold tolerant among all larval stages. Furthermore, *B. tau* at different life stages (eggs, larvae, pupae, and adults) nonetheless survived well within the adverse temperatures tested, ranging from −5 °C to 0 °C and 39 °C to 42 °C; hence, this pest harbors a wide temperature threshold range for survival, suggesting it can likely spread to and invade most temperate regions. In sum, our study provided timely, valuable information that advances our understanding of acute cold and heat tolerance in *B. tau*.

Cold tolerance is a critical factor determining the survival and distribution of invasive insects in temperate and cold regions [13,14,18,21,26]. Generally, the quantification of an insects’ cold tolerance is usually performed by preliminary measurements of their SCP [18,21,26,33]. For insects, the lower their SCP, the higher their survival probability in the face of cold stress conditions [34,35]. The SCP of insects varies greatly across different developmental stages. For instance, the SCPs of *Carpomya vesuviana* (Costa) and *Bactrocera cucurbitae* (Coquillett) are lower at their pupal stage than larval stage [36,37]. Similarly, for *Bactrocera dorsalis* (Hendel), its pupae have the lowest SCP among all the developmental stages, followed by the adults and larvae [38]. Likewise, we uncovered significant differences in the SCPs across life stages of *B. tau*, in that those of larvae, pupae, and adults of *B. tau* varied from −13.36 to −7.07 °C, −22.95 to −9.77 °C, and −15.69 to −7.69 °C, respectively, indicating a stronger cold-tolerance ability of pupae than in adults or larvae. Significant differences among the SCPs among the developmental stages of an insect may drive by various internal factors, such as life stage, body size, nutritional and physiological status, and the contents of various metabolites [39,40,41]. Furthermore, the SCP values of *B. tau* eggs were the lowest among all life stages, perhaps conferred by their small size and inactivity, as well as the protective shell of the egg structure, which may contain antifreeze-like substances [42,43,44]. Specifically, previous studies have classified the cold tolerance of insects into five categories: freeze intolerant, freeze tolerant, chill susceptible, chill tolerant and opportunistic survival [20,45]. Chill susceptible insects are characterized by extensive supercooling capacity that allows them to survive moderate low temperature exposure, but high mortality can occur after brief exposure to temperatures substantially above the SCP. Thus, SCP is not a reliable indicator of cold tolerance in chill susceptible insects [20,45]. As a matter of fact, several life stages of *B. tau* displayed significant mortality when exposed at temperatures higher than their mean SCP. This pest can be considered chill susceptible. In this context, the determination of SCP is necessary, but not sufficient for characterizing the cold tolerance of *B. tau*.

Generally, insects undergo complex ecological and environmental changes throughout their life history, wherein their physiological functions, basic behaviors, adaptability, and even their evolutionary pathways, are influenced and controlled by ambient temperature [12,13,14,29]. To date, most studies have focused on the influence of average temperature fluctuations on insects’ adaptability, developmental rates, fertility, and ensuing population dynamics and geographical distributions. Nevertheless, predictions based on population models that rely on average temperatures may lead to an overestimation of the optimum temperature, safe temperature ranges, and fitness for most insect species [46,47,48,49]. In particular, the profound effect of extreme temperature upon the viability and fitness of insects is more important than that of average temperature [3,15,25]. For instance, the population growth of *Bactrocera dorsalis* and *B. cucurbitae* is reduced under extreme high temperatures, and this limits their establishment more than do lower temperatures [50]. When subjected to acute low or high temperature stress, insects usually show outstanding cold or heat tolerance, which generates, inter alia, population variation, seasonal variation, and geographical variation in their adaptive traits [51,52]. Thus, the determination of survivorship at extreme low or high temperatures is paramount to evaluating the cold or heat tolerance capability of insects [14,21,26]. In cold winters, *B. tau* individuals often encounter a sudden drawdown of temperature for short- or long-term periods, and it is inevitable for them to experience acute heat stress during the summer in tropical and subtropical regions [3]. Here, we found that the survival rate of *B. tau* gradually decreased in all life stages with corresponding decreases or increases in the tested temperatures. The egg stage was the most sensitive to increased temperatures but the least sensitive to decreased temperatures. Among larval stages, it was 3rd instar larvae that were the most tolerant stage to either acute heat or cold treatments. This result is somewhat consistent with Mwando et al. [53] who reported that 3rd larva of *B. dorsalis* was the most heat tolerant among that insect’s immature stages. Furthermore, after being exposed to −9 °C or 43 °C for 4 h, the survival rate of each developmental stage of *B. tau* converged to their lowest level, that of nil (zero percentage). These results concur with those obtained by Huang et al. [3] in which *B. tau* was found unable to survive at temperatures above 42 °C. Hopefully, such basic knowledge of *B. tau*’s temperature-dependent mortality is essential to design effective phytosanitary protocols for this pest. For *B. tau*, it is commonly transported in all stages-eggs, larvae, or pupae, and usually within the host fruits or inside packages [2]. Phytosanitary disinfestation treatments are used to prevent the introduction and spread of this fruit fly into new countries or areas [2,54]. Our current study suggested that consignments of suspected fruit fly infestations may be quarantined by using acute low or heat temperature, such as exposure to −9 °C or 43 °C for 4 h to kill all life-stages of this pest.

Notably, the survival of *B. tau* at each developmental stage was jointly influenced by temperature stress intensity and the exposure duration. Almost 40 years ago, Turnock et al. [55] proposed a simple conceptual framework to describe different temperature zones within the thermobiological span of invertebrates. In the cold injury zone, both survival and development are negatively affected by low temperature [16]. The three-way mortality–temperature–time relationship could also be used to calculate the ULCIZ, the temperature above which cold does not cause mortality even after an ecologically meaningful period of time [35,56]. Our results showed that eggs of *B. tau* have the lowest ULCIZ (−2.819 °C), followed by 3-day-old adults (ULCIZ = −2.513 °C) and 1-day-old pupae (ULCIZ = −2.504 °C). Meanwhile, according to the concept and formula of ULCIZ, we calculated preliminary values for the LLTIZ, i.e., the temperature above which mortality ensues after an ecologically meaningful period of time. The 3-day-old larvae harbor a greater heat tolerance (LLTIZ = 41.443 °C) than newly hatched larvae (LLTIZ = 39.805 °C), with a similar phenomenon observed in *Plutella xylostella* L. [57]. Eggs of *B. tau* were the least tolerant of heat (LLTIZ = 37.144 °C), while 3-day-old adults (LLTIZ = 39.394 °C) exhibited slightly higher heat tolerance than 1-day-old pupae (LLTIZ = 38.285 °C). Coincidentally, those model-predicted values corroborate well with our empirical observations in the cold and heat tolerance assays. In this context, our results demonstrate that *B. tau*’s tolerance to acute cold and heat stress could be accurately quantified and compared by using these two parameters alone (i.e., ULCIZ, LLTIZ).

## 5. Conclusions

This study’s findings further enrich our understanding of the tolerance of *B. tau* to extreme cold and heat stress, and therefore, could be used as a basis for this pest’s post-harvest phytosanitary treatment. This is especially relevant, given that the most suitable development and reproduction temperature for *B. tau* is from 25 °C to 31 °C under laboratory conditions [3,58]. Nevertheless, the experimental population of *B. tau* we used was cultured in our lab under constant conditions in a stable environmental temperature at 25 ± 1 °C, whereas *B. tau* actually grows and develops under varying temperature environments in the field. In this context, determining the tolerance range of wild populations of *B. tau* to extreme temperature stress, and the physiological and molecular mechanisms enabling its cold or heat tolerance responses, should be explored in future studies.

## Figures and Tables

**Table 1:** Mean supercooling point for different developmental stages of *Bactrocera tau*.

Developmental Stages	Supercooling Points (SCPs)	
	Mean SCP ± SE (°C)	Range (°C) (Min., Max.)
Egg	−25.82 ± 0.51	−28.1, −14.8
1st instar larvae	−7.07 ± 0.61	−13.0, −1.1
2nd instar larvae	−12.13 ± 0.93	−20.3, −4.6
3rd instar larvae	−13.36 ± 0.87	−22.0, −4.0
1-day-old pupae	−9.77 ± 0.49	−13.2, −3.5
2-day-old pupae	−11.63 ± 0.78	−21.0, −2.5
3-day-old pupae	−16.69 ± 0.66	−20.9, −3.9
4-day-old pupae	−18.22 ± 0.70	−26.4, −11.9
5-day-old pupae	−18.97 ± 0.88	−27.1, −8.0
6-day-old pupae	−22.95 ± 0.34	−26.1, −18.8
7-day-old pupae	−19.78 ± 0.60	−25.4, −2.2
8-day-old pupae	−18.79 ± 0.47	−23.7, −12.3
2–3-day-old female adult	−7.92 ± 0.49	−13.7, −3.8
2–3-day-old male adult	−9.99 ± 0.83	−18.5, −1.8
8–10-day-old female adult	−14.63 ± 0.78	−22.0, −4.1
8–10-day-old male adult	−15.69 ± 0.90	−25.5, −7.4
25–30-day-old female adult	−11.35 ± 1.01	−20.3, −4.4
25–30-day-old male adult	−12.92 ± 0.72	−20.7, −0.4
45–50-day-old female adult	−7.69 ± 0.72	−23.6, −3.7
45–50-day-old male adult	−11.01 ± 0.79	−15.8, −2.5
65–70-day-old female adult	−8.77 ± 0.76	−16.2, −2.3
65–70-day-old male adult	−8.81 ± 0.69	−19.7, −3.8
85–90-day-old female adult	−8.36 ± 0.63	−15.2, −2.8
85–90-day-old male adult	−9.04 ± 0.87	−18.6, −3.1
105–110-day-old female adult	−8.72 ± 0.65	−16.6, −3.0
105–110-day-old male adult	−9.78 ± 0.96	−22.1, −2.3

**Table 2:** Predicted temperature required to achieve 50% mortality of different life stages of *Bactrocera tau* under different cold treatments.

Life Stages	The Semi-Lethal Temperature (LLT_50_) (°C)			
	0.5 h	1 h	2 h	4 h
Egg	−16.39	−13.96	−4.66	−2.40
1st instar larvae	−4.88	−3.15	−1.68	−0.86
2nd instar larvae	−4.23	−3.39	−1.84	−1.43
3rd instar larvae	−4.43	−3.87	−1.97	−1.62
1-day-old pupae	−5.27	−2.60	−2.11	−1.51
3-day-old adults	−3.69	−2.46	−1.80	−1.44

**Table 3:** Predicted LLT_50_ (°C) of different life stages of *Bactrocera tau* with different exposure heat temperatures and durations.

Life Stages	The Semi-Lethal Temperature (LLT_50_) (°C)			
	0.5 h	1 h	2 h	4 h
Egg	39.99	37.43	31.51	27.35
1st instar larvae	41.15	40.91	39.61	39.41
2nd instar larvae	41.92	40.99	40.74	40.28
3rd instar larvae	45.23	41.83	41.46	40.74
1-day-old pupae	43.90	42.69	40.74	38.97
3-day-old adults	42.63	41.06	40.48	38.22

## Data Availability

All data sets presented in this study are included in the article and can be availed by the authors upon reasonable request.

## References

[1] Jaleel W., Lu L., He Y. (2018). Biology, taxonomy, and IPM strategies of *Bactrocera tau* Walker and complex species (Diptera; Tephritidae) in Asia: A comprehensive review. Environ. Sci. Pollut. R..

[2] Huang Y., Gu X., Peng X., Tao M., Peng L., Chen G., Zhang X. (2020). Effect of short-term low temperature on the growth, development, and reproduction of *Bactrocera tau* (Diptera: Tephritidae) and *Bactrocera cucurbitae*. J. Econ. Entomol..

[3] Huang Y., Gu X., Peng X., Tao M., Chen G., Zhang X. (2020). Effect of short-term high-temperatures on the growth, development and reproduction in the fruit fly, *Bactrocera tau* (Diptera: Tephritidae). Sci. Rep..

[4] Vasudha A., Ahmad M.A., Agarwal M.L. (2019). Life history traits and immature stages of *Zeugodacus (Zeugodacus) tau* (Walker) (Diptera: Tephritidae). J. Entomol. Zool. Stud..

[5] An K.P., Wu B.F., Shen K., Zhang R.J. (2011). Charateristic and research progress on control technology of pumpkin fruit fly *Bactrocera tau* (Walker) (Diptera: Tephritidae). J. Chang. Veg..

[6] Li X., Yang H., Hu K., Wang J. (2020). Temporal dynamics of *Bactrocera (Zeugodacus) tau* (Diptera: Tephritidae) adults in north Jiangxi, a subtropical area of China revealed by eight years of trapping with cuelure. J. Asia-Pac. Entomol..

[7] Shi W., Kerdelhué C., Ye H. (2014). Genetic structure and colonization history of the fruit fly *Bactrocera tau* (Diptera: Tephritidae) in China and southeast Asia. J. Econ. Entomol..

[8] Khalid M. (1999). Taxonomy of the *Bactrocera (Zeugodacus) tau* (Tephritidae: Diptera) complex in Asia. Pak. J. Zool..

[9] Hasyim A., Muryati M., Kogel D. (2008). Population fluctuation of adult males of the fruit fly, *Bactrocera tau* Walker (Diptera: Tephritidae) in passion fruit orchards in relation to abiotic factors and sanitation. Indones. J. Agr. Sci..

[10] Ma C.S., Ma G., Zhao F. (2014). Impact of global warming on cereal aphids. Chin. J. Appl. Entomol..

[11] Izadi H., Mohammadzadeh M., Mehrabian M. (2019). Cold tolerance of the *Tribolium castaneum* (Coleoptera: Tenebrionidae), under different thermal regimes: Impact of cold acclimation. J. Econ. Entomol..

[12] Enriquez T., Colinet H. (2017). Basal tolerance to heat and cold exposure of the spotted wing drosophila, *Drosophila suzukii*. PeerJ.

[13] Pei J., Li C., Ren L., Zong S. (2020). Factors influencing cold hardiness during overwintering of *Streltzoviella insularis* (Lepidoptera: Cossidae). J. Econ. Entomol..

[14] Iqbal J., Zhang X.X., Chang Y.W., Du Y.Z. (2021). Differential response of leafminer flies *Liriomyza trifolii* (Burgess) and *Liriomyza sativae* (Blanchard) to rapid cold hardening. Insects.

[15] Bawa S.A., Gregg P.C., Del Soccoro A.P., Miller C., Andrew N.R. (2021). Estimating the differences in critical thermal maximum and metabolic rate of *Helicoverpa punctigera* (Wallengren) (Lepidoptera: Noctuidae) across life stages. PeerJ.

[16] Colinet H., Sinclair B.J., Vernon P., Renault D. (2015). Insects in fluctuating thermal environments. Annu. Rev. Entomol..

[17] Liu Z., Gong P., Wu K., Wei W., Sun J., Li D. (2007). Effects of larval host plants on over-wintering preparedness and survival of the cotton bollworm, *Helicoverpa armigera* (Hübner) (Lepidoptera: Noctuidae). J. Insect Physiol..

[18] Li C., Pei J., Li J., Liu X., Ren L., Luo Y. (2021). Overwintering larval cold tolerance of *Sirex noctilio* (Hymenoptera: Siricidae): Geographic variation in northeast China. Insects.

[19] Noor-Ul-Ane M., Jung C. (2021). Characterization of cold tolerance of immature stages of small hive beetle (SHB) *Aethina tumida* Murray (Coleoptera: Nitidulidae). Insects.

[20] Andreadis S.S., Athanassiou C.G. (2017). A review of insect cold hardiness and its potential in stored product insect control. Crop Prot..

[21] Pourani M.S., Mahdian K., Izadi H., Basirat M., Sahhafi S.R. (2019). Cold tolerance and supercooling points of two ladybird beetles (Col.: Coccinellidae): Impact of the diet. Cryobiology.

[22] Ditrich T. (2018). Supercooling point is an individually fixed metric of cold tolerance in *Pyrrhocoris apterus*. J. Therm. Biol..

[23] Worland M.R., Convey P., Luke ov A. (2000). Rapid cold hardening: A gut feeling. Cryo Lett..

[24] Terblanche J.S., Hoffmann A.A., Mitchell K.A., Rako L., Le Roux P.C., Chown S.L. (2011). Ecologically relevant measures of tolerance to potentially lethal temperatures. J. Exp. Biol..

[25] Pieterse W., Terblanche J.S., Addison P. (2017). Do thermal tolerances and rapid thermal responses contribute to the invasion potential of *Bactrocera dorsalis* (Diptera: Tephritidae)?. J. Insect Physiol..

[26] Bastola A., Davis J.A. (2018). Cold tolerance and supercooling capacity of the redbanded stink bug (Hemiptera: Pentatomidae). Environ. Entomol..

[27] Jing X.H., Kang L. (2002). Research progress in insect cold hardiness. Acta Ecol. Sin..

[28] Dou Q.C., Liu X.F., Yao W.F., Ye H. (2011). Tolerance of adult guava fruit fly (Diptera: Tephritidae) to low temperature. Southwest China J. Agric. Sci..

[29] Kirk Green C., Moore P.J., Sial A.A. (2019). Impact of heat stress on development and fertility of *Drosophila suzukii* Matsumura (Diptera: Drosophilidae). J. Insect Physiol..

[30] Eben A., Reifenrath M., Briem F., Pink S., Vogt H. (2018). Response of *Drosophila suzukii* (Diptera: Drosophilidae) to extreme heat and dryness. Agric. Forest Entomol..

[31] Mutamiswa R., Machekano H., Chidawanyika F., Nyamukondiwa C. (2019). Life-stage related responses to combined effects of acclimation temperature and humidity on the thermal tolerance of *Chilo partellus* (Swinhoe) (Lepidoptera: Crambidae). J. Therm. Biol..

[32] Wang H., Ma Z., Cui F., Wang X., Guo W., Lin Z., Yang P., Kang L. (2012). Parental phase status affects the cold hardiness of progeny eggs in locusts. Funct. Ecol..

[33] Mohammadzadeh M., Izadi H. (2018). Cooling rate and starvation affect supercooling point and cold tolerance of the khapra beetle, *Trogoderma granarium* Everts fourth instar larvae (Coleoptera: Dermestidae). J. Therm. Biol..

[34] Nedved O., Hodkova M., Brunnhofer V., Hodek I. (1995). Simultaneous measurement of low temperatures survival and supercooling in a sample of insects. Cryo Lett..

[35] Hodkova M., Hodek I. (1997). Temperature regulation of supercooling and gut nucleation in relation to diapause of *Pyrrhocoris apterus* (L.) (Heteroptera). Cryobiology.

[36] Ding J.T., Adil S., Cheng X.T., Huang X.Y., Amina (2014). Supercooling points and freezing points of *Carpomya vesuviana* Costa. Acta Agr. Boreali-Occident. Sin..

[37] Huang N.N., Dai P., Fu Y.G., Huang Q.Y. (2015). Measurement of physiological indices of cold tolerance in *Bactrocera cucurbitae* (Coquillett). Chin. J. Appl. Entomol..

[38] Hou B.H., Zhang R.J. (2007). Supercooling capacity of the Oriental fruit fly, *Bactrocera dorsalis* (Hendel) (Diptera: Tephritidae). Entomol. Sin..

[39] Kost’ál V., Slachta M., Simek P. (2001). Cryoprotective role of polyols independent of the increase in supercooling capacity in diapausing adults of *Pyrrhocoris apterus* (Heteroptera: Insecta). Comp. Biochem. Physiol. B Biochem. Mol. Biol..

[40] Worland M.R., Leinaas H.P., Chown S.L. (2006). Supercooling point frequency distributions in Collembola are affected by moulting. Funct. Ecol..

[41] Mohammadzadeh M., Izadi H. (2018). Cold acclimation of *Trogoderma granarium* everts is tightly linked to regulation of enzyme activity, energy content, and ion concentration. Front. Physiol..

[42] Wang S.Z., Li Q., Feng C.H., Zhang M., Jiang F., Yang G., Luo L.M. (2007). Cold-hardiness of *Locusta migratoria tibetensis* in each developmental stage. Chin. Bull. Entomol..

[43] Tang Z.Z., Wang J., Liu X.R., Li Y.Q., Lv B.Q., Peng Z.Q., Yu Y.H., Liu M.W. (2016). Comparison of cold tolerance in two tropical invasive pests. Plant Quar..

[44] Ditrich T., Koštál V. (2011). Comparative analysis of overwintering physiology in nine species of semi-aquatic bugs (Heteroptera: Gerromorpha). Physiol. Entomol..

[45] Bale J.S. (1996). Insect cold hardiness: A matter of life and death. Eur. J. Entomol..

[46] Parmesan C., Yohe G. (2003). A globally coherent fingerprint of climate change impacts across natural systems. Nature.

[47] Cannon R.J. (1998). The implications of predicted climate change for insect pests in the UK, with emphasis on non-indigenous species. Global. Chang. Biol..

[48] Ragland G.J., Kingsolver J.G. (2008). The effect of fluctuating temperatures on ectotherm life-history traits: Comparisons among geographic populations of *Wyeomyia smithii*. Evol. Ecol. Res..

[49] Rall B.C., Vucic-Pestic O., Ehnes R.B., Emmerson M., Brose U. (2010). Temperature, predator-prey interaction strength and population stability. Global. Chang. Biol..

[50] Vargas R.I., Walsh W.A., Kanehisa D., Stark J.D., Nishida T. (2000). Comparative demography of three Hawaiian fruit flies (Diptera: Tephritidae) at alternating temperatures. Ann. Entomol. Soc. Am..

[51] Gibert P., Moreteau B., Pétavy G., Karan D., David J.R. (2001). Chill-coma tolerance, a major climatic adaptation among *Drosophila* species. Evolution.

[52] Hoffmann A.A., Sørensen J.G., Loeschcke V. (2003). Adaptation of *Drosophila* to temperature extremes: Bringing together quantitative and molecular approaches. J. Therm. Biol..

[53] Mwando N.L., Ndlela S., Meyhöfer R., Subramanian S., Mohamed S.A. (2021). Hot water treatment for post-harvest disinfestation of *Bactrocera dorsalis* (Diptera: Tephritidae) and its effect on cv. tommy atkins mango. Insects.

[54] Follett P.A., Manoukis N.C., Mackey B. (2018). Comparative cold tolerance in *Ceratitis capitata* and *Zeugodacus cucurbitae* (Diptera: Tephritidae). J. Econ. Entomol..

[55] Turnock W.J., Lamb R.J., Bodnaryk R.P. (1983). Effects of cold stress during pupal diapause on the survival and development of *Mamestra configurata* (Lepidoptera: Noctuidae). Oecologia.

[56] Nedved O. (1998). Modelling the relationship between cold injury and accumulated degree days in terrestial arthropods. Cryo Lett..

[57] Zhang W., Chang X.Q., Hoffmann A., Zhang S., Ma C.S. (2015). Impact of hot events at different developmental stages of a moth: The closer to adult stage, the less reproductive output. Sci. Rep..

[58] Zhou C.Q., Wu K.K., Chen H.D., Yang P.J., Dowell R.V. (1994). Effect of temperature on the population growth of *Bactrocera tau* (Walker) (Dipt., Tephritidae). J. Appl. Entomol..

